# Supplementary material for: Correction: Vibrotactile information improves proprioceptive reaching target localization
Source: PLoS One. 2018 Oct 25;13(10):e0206574. doi: 10.1371/journal.pone.0206574 (PMC6201965; doi:10.1371/journal.pone.0206574)
Supplement: S2 File — (PDF) [file pone.0206574.s002.pdf]

PLOS ONE

RESEARCH ARTICLE

Vibrotactile information improves proprioceptive reaching target localization

Laura Mikula[1,2], Sofia Sahnoun[2], Laure Pisella[1], Gunnar Blohm[3], Aarlenne Zein Khan[2]*

1 Centre de Recherche en Neurosciences de Lyon (CRNL), ImpAct team, Inserm U1028, CNRS UMR 5292, University Claude Bernard Lyon 1, Bron, France, **2** School of Optometry, University of Montreal, Montréal, Québec, Canada, **3** Centre for Neuroscience Studies, Queen's University, Kingston, Ontario, Canada

* aarlenne.khan@umontreal.ca

Check for updates

OPEN ACCESS

Citation: Mikula L, Sahnoun S, Pisella L, Blohm G, Khan AZ (2018) Vibrotactile information improves proprioceptive reaching target localization. PLoS ONE 13(7): e0199627. https://doi.org/10.1371/journal.pone.0199627

Editor: Christos Papadelis, Boston Children's Hospital / Harvard Medical School, UNITED STATES

Received: February 5, 2018

Accepted: June 11, 2018

Published: July 6, 2018

Copyright: © 2018 Mikula et al. This is an open access article distributed under the terms of the Creative Commons Attribution License, which permits unrestricted use, distribution, and reproduction in any medium, provided the original author and source are credited.

Data Availability Statement: Files are available from the Open Science Framework platform (https://osf.io/c3ztu/).

Funding: LM received support from a PhD excellence scholarship from Faculté des Etudes Supérieures et Postdoctorales and École d'Optométrie de l'Université de Montréal. AZK and GB were funded by the Natural Sciences and Engineering Research Council of Canada (http://www.nserc-crsng.gc.ca/index_eng.asp). AZK was additionally supported by the Canada Research

Abstract

When pointing to parts of our own body (e.g., the opposite index finger), the position of the target is derived from proprioceptive signals. Consistent with the principles of multisensory integration, it has been found that participants better matched the position of their index finger when they also had visual cues about its location. Unlike vision, touch may not provide additional information about finger position in space, since fingertip tactile information theoretically remains the same irrespective of the postural configuration of the upper limb. However, since tactile and proprioceptive information are ultimately coded within the same population of posterior parietal neurons within high-level spatial representations, we nevertheless hypothesized that additional tactile information could benefit the processing of proprioceptive signals. To investigate the influence of tactile information on proprioceptive localization, we asked 19 participants to reach with the right hand towards the opposite unseen index finger (proprioceptive target). Vibrotactile stimuli were applied to the target index finger prior to movement execution. We found that participants made smaller errors and more consistent reaches following tactile stimulation. These results demonstrate that transient touch provided at the proprioceptive target improves subsequent reaching precision and accuracy. Such improvement was not observed when tactile stimulation was delivered to a distinct body part (the shoulder). This suggests a specific spatial integration of touch and proprioception at the level of high-level cortical body representations, resulting in touch improving position sense.

Introduction

To execute a hand reaching movement, the central nervous system needs to localize the target with respect to the hand. Its position can be derived from inputs provided by one or multiple sensory modalities such as vision, audition or somatosensation. Multisensory integration is referred to as the combination of information arising from different sensory modalities to form a unified and coherent representation of our environment and body. Accordingly, the brain combines all the relevant sensory information about the object of interest in order to decrease the variance (the uncertainty) and build a more reliable representation of that object

Chair program (http://www.chairs-chaires.gc.ca/home-accueil-eng.aspx). LP was supported by the Centre National de la Recherche Scientifique and the Laboratoires d'excellence and the Initiatives d'excellence (ANR-11-LABX-0042). The funders had no role in study design, data collection and analysis, decision to publish, or preparation of the manuscript.

Competing interests: The authors have declared that no competing interests exist.

[1,2]. Indeed, it has been shown that spatial localization was less variable for visual-auditory targets than for targets specified by vision or audition only [3,4]. These findings suggest that the more sensory information available about the target, the more accurate its estimate.

When pointing to unseen parts of our own body (e.g. the opposite index finger), the position of the target is derived from proprioceptive signals. Proprioception corresponds to the sense of our body position in space. Consistent with the principles of multisensory integration, it has been found that participants better matched the position of their index finger when they could see their opposite arm during movement than when being blindfolded during the task [5,6]. The localization of the fingertip was more precise in the presence of both vision and proprioception than when using visual or proprioceptive signals only. These results provide evidence that fingertip localization can be more precise if another sensory modality, in addition to proprioception, provides further information about the finger position.

Unlike vision, touch may not provide additional information about finger position in space, since fingertip tactile information theoretically remains the same irrespective of the postural configuration of the upper limb. However, touch can be regarded as a possible source of additional information for position sense, since touch and proprioception, although considered as separate modalities, have been shown to closely interact with each other. Behavioral studies have shown that tactile perception can be modulated by changes in proprioceptive signals, induced by active changes in hand posture [7] or tendon vibration [8]. Conversely, a finger-position matching task has been reported to be affected by nerve block and cutaneous anesthesia [9], indicating that cutaneous afferents may provide a crude position sense for the fingers. Moreover, it has been shown that the localization of a proprioceptive target (i.e., the fingertip) was improved when participants contacted a surface with their target fingertip, which provides them with tactile feedback [10–12]. Similarly, accuracy in pointing movements was enhanced when endpoint contact occurred with the effector fingertip [13]. In contrast, digital anesthesia resulted in impaired fingertip localization [11] as well as decreased movement accuracy during typing [14]. This relationship between touch and proprioception is likely to be explained by the convergence of proprioceptive and tactile signals at the cortical level; electrophysiological recordings in monkey have shown that neurons in the hand representation of the primary somatosensory cortex code both tactile and proprioceptive modalities during a reach-to-grasp task [12,15]. It has also been established that neurons in the somatosensory cortex have both cutaneous and proprioceptive receptive fields [16,17]. Taken together, these findings suggest that tactile afferent information may contribute to proprioception and improve the accuracy of the hand proprioceptive estimate.

The skin contains several mechanoreceptors, including Meissner and Pacinian corpuscles. Meissner corpuscles are located in the superficial layers of the skin and are sensitive to light touch while Pacinian corpuscles are found in deeper layers and respond to deep skin pressure and vibration. The properties of these two receptors suggest that they might be activated by fingertip contact; Pacinian and Meissner corpuscles are fastadapting receptors which are both sensitive to abrupt but not sustained stimuli [18], such as when a finger makes or breaks contact with an object. Therefore, it is difficult to distinguish the relative contributions of Pacinian and Meissner corpuscules to the enhancement of proprioception following fingertip contact with a surface [10–13]. However, these two types of mechanoreceptors show different responses to cutaneous vibrations. Meissner corpuscles respond to low frequencies, 10–80 Hz, whereas Pacinian corpuscles are sensitive to vibrations at higher frequencies, 80–450 Hz [19]. Consequently, by stimulating either of these receptors, it would be possible to know which one contributes to the enhancement of proprioceptive localization.

It has been shown that the ability to detect flexion and extension movements imposed at the interphalangeal joints of a finger was impaired when 300 Hz vibrations were applied to the

adjacent or the test digit. In contrast, vibrotactile stimuli at 30 Hz did not alter proprioception in the finger [20,21]. The detection of passive finger movements at the interphalangeal joints is thus impaired by the specific activation of Pacinian, but not Meissner, afferents. These results demonstrate that vibrotactile stimulation can modulate proprioceptive acuity in a passive perceptual task, in which no action is involved. However, to our knowledge this has not been tested in a motor task, such as reaching, where the target location corresponds to the position of the fingertip.

The goal of the present study was to investigate the influence of vibrotactile information on the proprioceptive localization of the finger in a motor context. To this purpose, we asked participants to perform reaches to proprioceptively defined targets. They reached with the right index finger (reaching finger) to the unseen left index finger (target finger), which was passively displaced to different locations. Tactile vibrations at 30 or 300 Hz were delivered to the target index fingertip prior to movement onset. When vibrations are applied, the left index finger receives tactile information, in addition to existing proprioceptive information, about its location in space. In order to reach accurately, we presume that the brain constructs a reliable estimate of the target finger position using all the sensory information available. As suggested by previous studies [19– 21], high- and low-frequency vibrations are more likely to activate Pacinian and Meissner corpuscles, respectively. We thus used 30 and 300 Hz vibrotactile stimulations to determine if one of these two mechanoreceptors contribute more than the other to touch-proprioception integration, or whether they both contribute to finger proprioceptive localization. We measured reach endpoint accuracy and precision to assess the effect of vibrations. We found that vibrotactile stimulations delivered at low and high frequencies improved both accuracy and precision of finger localization in a proprioceptive reaching task. A control condition in which the vibration was applied elsewhere on the body showed that this improvement in proprioceptive localization cannot be attributed to a global arousal enhancement induced by the tactile stimulus.

Methods

Participants

Nineteen participants took part in this study (12 females, mean ± SD age = 25.3 ± 10.7 years). They were all right-handed, as assessed by the Edinburgh Handedness Inventory and all had normal or corrected-to-normal vision. Participants were administered a questionnaire to ensure that they did not suffer from neurological, sensory or motor deficits, which may have interfered with their performance. All gave informed written consent to participate in this experiment which conformed to the Declaration of Helsinki (2008) for experiments on human subjects. All experimental procedures were approved by the health research ethics committee in France (CPP Nord-Ouest I, Lyon, 2017-A02562-51) and at the University of Montreal (17-034-CERES-D).

Apparatus

Participants sat in a dark room on a height-adjustable chair in front of a slanted table. Their head was held steady on a chin rest, aligned with their body midline. A wide-screen OLED monitor (55 inches diagonal, 1920 x 1080 pixels, LG) was placed facing downwards above the table and a half-reflecting mirror was positioned in between the screen and the table so that the screen was projected onto the tabletop surface. The half-reflecting mirror prevented participants from seeing their hands unless there was light underneath the mirror; in that case vision of the hand was possible. Participants performed a proprioceptive pointing task. They were asked to reach with the right index finger (reaching finger) to the unseen left index finger

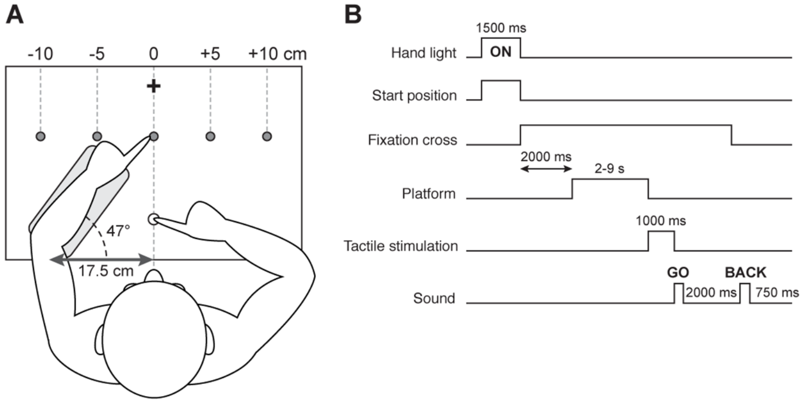

Fig 1. Schematic of the apparatus, top view (A) and sequence of a trial (B). (A) Participants' left forearm was resting on a motorized platform that could move laterally to the five proprioceptive target positions (grey circles). The left forearm was positioned at an angle of 47° approximately. When the left index finger was aligned with the central target, the left elbow was 17.5 cm left relative to the body midline. The start position (white circle) for the right index finger was 15 cm ahead of the participants' torso. The fixation cross (black cross) was located further than the proprioceptive target positions. (B) The hang light and the start position were first turned on for 1500 ms and participants were asked to align their right index finger with the start position. Then, the fixation cross appeared and participants maintained gaze on the cross until the end of the trial. After 2000 ms, the platform started moving the left target hand for a variable amount of time (between 2 and 9 s). Afterwards, a tactile vibration was delivered to the left index finger for 1000 ms. Then a first auditory tone served as a "go" signal for participants to start reaching with their right index finger towards their left index fingertip. After 2000 ms, a second auditory tone was presented and participants put their right index finger back to the start position. The next trial started 750 ms later.

https://doi.org/10.1371/journal.pone.0199627.g001

(target finger). Participants' left forearm was resting on a platform in such a way that when the left index finger was aligned with the body midline, the elbow was located on average 17.5 cm on the left relative to the center (Fig 1A). The left forearm was positioned at an angle of 47°. The forearm platform was motorized and could move laterally (left or right) to different target positions. The target locations for the left index finger were at -10, -5, 0, +5 and +10 cm with respect to the body midline. A tactor was positioned on the left index fingertip and connected to an amplifier (TactAmp 4.2, Dancer Design, England, United Kingdom) that delivered tactile vibrations at a frequency of either 30 or 300 Hz. Eye movements were monitored using an Eye-Link 1000 Plus (SR Research, Mississauga, Ontario, Canada) at a sampling rate of 1000 Hz. The positions of both left and right index fingers were measured using an Optotrak 3D Investigator recording system (NDI, Waterloo, Canada). This system recorded the position of two infrared emitting diodes, each one attached to the tip of each index finger. The movement of the infrared markers was tracked and sampled at a rate of 500 Hz.

Procedure

The sequence of a trial is depicted in Fig 1B. At the beginning of each trial, a light was switched on for 1500 ms so that participants could see their hands. At the same time, a red dot aligned with the body midline and 15 cm distant from the torso was displayed also for 1500 ms. The red dot served as a start position and participants were asked to align their right index fingertip with the red dot and to keep it in this position until they began reaching. They kept their right fingertip balanced in the air above the table (no surface contact). As soon as the start position disappeared, a white fixation cross was displayed and participants were required to fixate the cross until the end of the trial. The fixation cross was aligned with the body midline above the target positions. After two seconds, the motorized platform moved the left target index finger to one of the 5 possible target locations. To prevent participants from learning proprioceptive

target positions across trials, the platform made several back-and-forth movements (from 1 to 5) before stopping on a target location. Then, a vibrotactile stimulation was applied to the left target index fingertip for 1000 ms. Vibrations could be delivered at 0 (no vibration condition), 30 or 300 Hz. After the tactile stimulation, a first auditory tone signaled to the participants that they could begin reaching with their right hand. Participants had 2 s to complete their reach before a second auditory tone instructed them to return to the start position. Participants were instructed to reach to a location just above their left fingertip, pause in the air, then return to the start position. Specifically, participants were asked to reach to where they thought their left index fingertip was as accurately as possible and to avoid contacting their left target index finger with their right hand. To ensure that participants performed the task properly, they first did a practice block and were asked to report when finger-finger contact occurred during the experiment. The next trial began after 750 ms.

Each block was composed of 15 trials (3 vibration frequencies x 5 target positions). Each of the possible combinations of target and vibration frequency was presented in a random order. Each participant completed between 10 and 20 blocks to obtain at least 4 trials for each combination of target and vibration frequency.

To test for a possible effect of the fingertip vibration by arousal enhancement, participants performed 2 additional control blocks in which the location of the vibrotactile stimulus was varied. The trials were identical to those in the main experiment except that the vibration was delivered to the left shoulder. The order of the blocks (control and main experiments) was counterbalanced across participants.

Data analysis

In this proprioceptive reaching task, errors were defined as the difference between the positions of the left (target) index finger and the right (reaching) index finger at the end of the movement. Since the position of the target hand was varied in the horizontal axis, we only considered reaching errors in the *x*-direction. Errors in the *x*-direction were computed for each trial by subtracting the *x*-position of the target hand from the *x*position of the right-hand endpoint. The constant *x*-error was expressed in mm and corresponded to the mean error in the *x*-direction for each target; this measure provides an estimate of the accuracy of the localization of the fingertip position. We used dispersion error as a measure of reach precision [22]. The dispersion error corresponded to the surface area of the endpoints around each corresponding target, it was expressed in mm^2 and computed with the following formula: $SD_x \times SD_y \times \pi$. With SD_x and SD_y corresponding to the standard deviations of reach endpoints in the x- and y-direction, respectively. Dispersion error provides an estimate of the precision of the localization of the fingertip position. Constant *x*-errors and dispersion errors were first calculated for each participant, vibration condition and target position, then averaged across target positions and participants. To test the influence of the vibration frequency on constant *x*errors and dispersion errors, a one-way repeated measures ANOVA was performed for each type of error separately. Similarly, one-way repeated measures ANOVAs were performed on constant x-errors and dispersion errors for each of the two attention control experiments. Tukey HSD tests were used for post-hoc comparisons of the means.

The threshold for statistical significance was set at 0.05 for all analyses.

Results

Reach endpoints relative to the five possible proprioceptive target locations are depicted in Fig 2 for one participant. Endpoints are represented for all three experimental conditions: the no vibration condition, the 30 Hz and the 300 Hz vibration conditions (in red, green and blue,

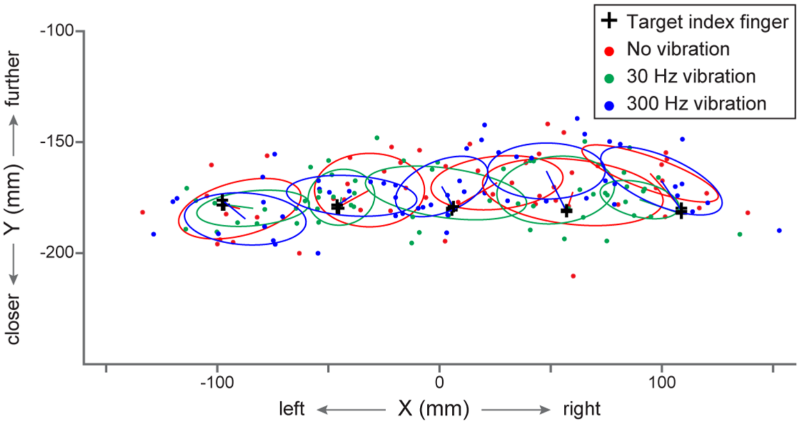

Fig 2. Reach endpoints for one participant. The participant reached in the dark with his right hand towards his unseen left target index finger under three conditions. A vibrotactile stimulation could be applied to the left index fingertip prior to movement onset at either 30 Hz (in green) or 300 Hz (in blue). Alternatively, no vibration was delivered (in red). Black crosses correspond to the average position of the target index finger to reach for. One-standard-deviation ellipses were computed for each target and each vibration condition. The center of the ellipses corresponds to the mean error in each condition.

https://doi.org/10.1371/journal.pone.0199627.g002

respectively). The one-standard-deviation ellipses correspond to the dispersion of reach endpoints and the center of ellipses represents the mean reach error. For this participant, reach endpoints were overall more scattered when no tactile stimulus was delivered to the target index fingertip (in red) compared to the 30 Hz and the 300 Hz vibration conditions (in green and blue).

Constant *x*-errors

A one-way repeated measures ANOVA was performed on the reach errors in the *x*direction. It revealed a significant effect of the vibration frequency ($F_{2,36}$ = 7.56, p = 0.002, η^2 = 41.30). Constant *x*-errors were equal to 12.3 ± 2.5 mm (mean ± SE) when no vibration was applied prior to the proprioceptive reach onset (Fig 3). Constant *x*-errors in the 30 Hz and the 300 Hz vibration conditions were 8.9 ± 2.1 mm and 9.1 ± 2.2 mm, respectively. Post-hoc tests showed that, compared to the no vibration condition, constant *x*-errors were significantly reduced when either a 30 Hz (mean ± SE of the difference = 3.4 ± 1.2 mm, t_{18} = 3.29, p = 0.004) or a 300 Hz vibration (3.2 ± 1.0 mm, t_{18} = 3.09, p = 0.006) was delivered to the left target index finger. However, constant *x*-errors were not significantly different between the 30 Hz and the 300 Hz vibration conditions (-0.2 ± 0.7 mm, t_{18} = 0.02, $p > 0.05$).

Dispersion errors

The one-way repeated measures ANOVA on the dispersion errors was also significant ($F_{2,36}$ = 4.61, p = 0.017, η^2 = 46.83). As depicted in Fig 4, the greatest dispersion errors are observed in the no vibration condition (923.1 ± 91.3 mm^2), followed by dispersion errors in the 30 Hz (819.7 ± 82.3 mm^2) and then in the 300 Hz vibration condition (805.6 ± 68.8 mm^2). Post-hoc tests showed that these errors significantly decreased when vibrotactile stimuli were delivered at 30 Hz (103.3 ± 41.6 mm^2, t_{18} = 2.10, p = 0.050) and 300 Hz (117.5 ± 44.0 mm^2, t_{18} = 2.49, p = 0.023). Dispersion errors between the 30 and 300 Hz vibration conditions did not significantly differ from each other (14.2 ± 37.6 mm^2, t_{18} = 0.08, $p > 0.05$).

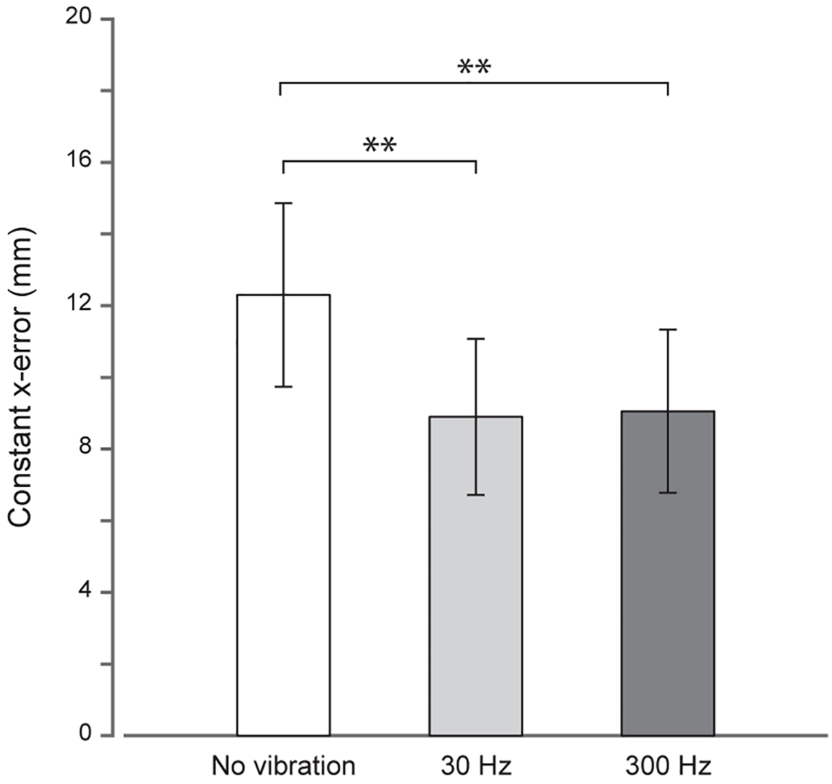

Fig 3. Constant *x*-errors (in mm) as a function of the vibrotactile stimulation applied. Errors when no vibration is applied to the left target index finger are represented by the white bar. Errors when 30 and 300 Hz vibrations are delivered are represented in light and dark grey bars, respectively. The error bars correspond to the standard error of the mean across participants. $^{**}p < 0.01$.

https://doi.org/10.1371/journal.pone.0199627.g003

Control experiment

We found that both high- and low-frequency vibrations applied to the target fingertip reduced constant *x*-errors and dispersion errors, suggesting that tactile information was combined with proprioception and improved spatial localization of the left target finger. It could be due to an effect of the vibration by arousal enhancement. To test for this, we investigated whether constant *x*-errors and dispersion errors changed when the vibration was delivered elsewhere. Thus, participants performed a control experiment where the vibrotactile stimulus was applied to the left shoulder. If reduced errors consecutive to the vibration of the left fingertip result from an effect of arousal, they should also be observed in this control condition. If they rather result from a specific spatial multi-sensory integration, then stimulation on the shoulder should not improve constant or dispersion errors compared to the no vibration condition.

The constant *x*-errors and the dispersion errors when the vibration was applied on the left shoulder are shown in Fig 5A and 5B, respectively. Constant *x*-errors in the no, 30 Hz and 300 Hz vibration conditions were equal to 8.1 ± 3.5 mm, 13.4 ± 3.1 mm and 11.3 ± 2.8 mm, respectively (Fig 5A). The one-way repeated measures ANOVA on the constant *x*-errors showed that the vibration frequency effect was significant ($F_{2,36} = 3.7$, $p = 0.035$, $\eta^2 = 41.30$). Post-hoc tests showed that constant *x*-errors were specifically increased when vibrotactile stimulation was delivered at 30 Hz (-5.2 ± 2.2 mm, $t_{18} = 2.39$, $p = 0.028$) and not when delivered at 300 Hz (-3.2 ± 1.8 mm, $t_{18} = 1.22$, $p > 0.05$). However, constant *x*-errors were not different between low- and high-frequency vibrotactile stimulations (2.0 ± 1.6 mm, $t_{18} = 0.61$, $p > 0.05$). As for

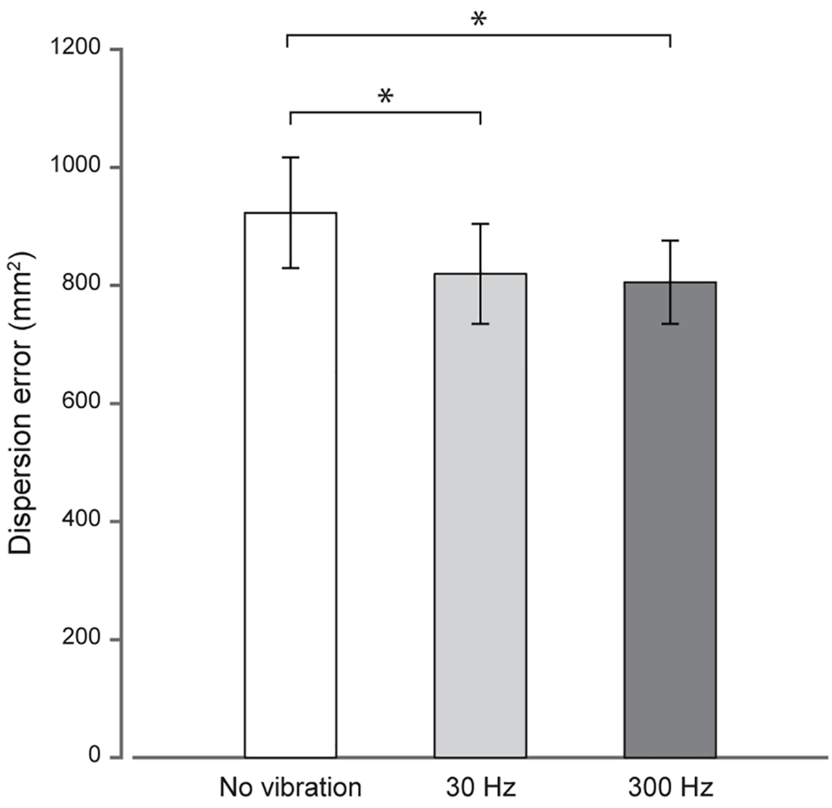

Fig 4. Dispersion errors (in mm^2) as a function of the vibrotactile stimulation applied. Errors when no vibration is applied to the left target index finger are represented by the white bar. Errors when 30 and 300 Hz vibrations are delivered are represented in light and dark grey bars, respectively. The error bars correspond to the standard error of the mean across participants. $^*p < 0.05$.

https://doi.org/10.1371/journal.pone.0199627.g004

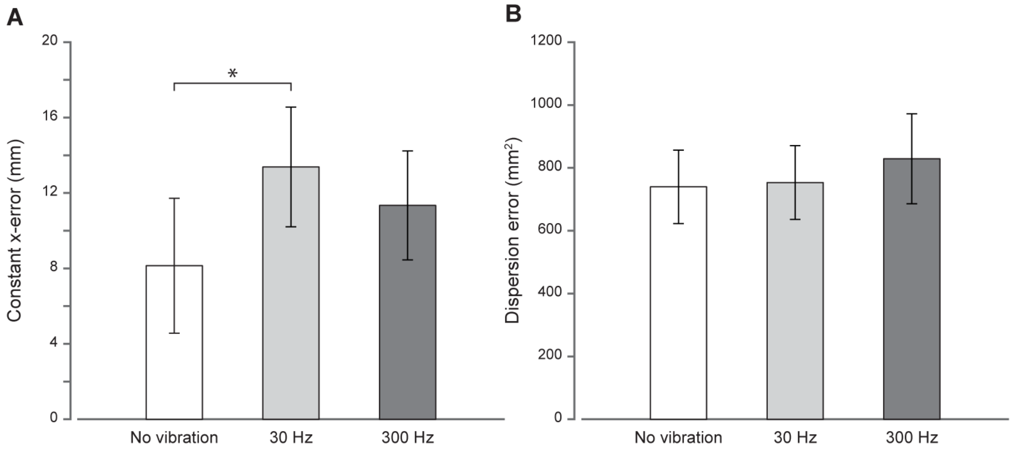

Fig 5. Constant *x*-errors (A) and dispersion errors (B) for the control experiment. In this experiment, vibrotactile stimulations are delivered to the left shoulder. Errors when no vibration is applied are represented by the white bar. Errors when 30 and 300 Hz vibrations are delivered are represented in light and dark grey bars, respectively. The error bars correspond to the standard error of the mean. (A) Constant *x*-errors (in mm) as a function of the vibrotactile stimulation applied to the left shoulder. (B) Dispersion errors (in mm^2) as a function of the vibrotactile stimulation applied to the left shoulder. $^*p < 0.05$.

https://doi.org/10.1371/journal.pone.0199627.g005

dispersion errors, there was no significant effect of vibration frequency when the left shoulder was stimulated ($F_{2,36}$ = 0.26, $p > 0.05$; Fig 5B). These findings suggest that the improved spatial localization of the left target finger following vibrotactile stimulus on the fingertip is unlikely due to global arousal effect of the vibration.

Discussion

In the context of multisensory information, it is acknowledged that the brain combines all the available sensory information to build a precise and robust representation of the world [1,23]. For instance, accurate reaching movements require precise target localization prior to motor execution and several studies have shown that this localization was better when more than one sensory modality provided information about the target position [3,4,24]. When pointing to our body parts (e.g. the opposite index finger), the target is proprioceptively defined and reaches tend to be more variable than those directed to visual targets [25]. This might be related to a greater uncertainty in the localization of proprioceptive versus visual targets [26]. Hence, proprioceptive reaching might be improved if a second sensory modality provides additional information about the spatial location of the target. The interaction between touch and proprioception that has been reported in previous studies [7–9] suggests that tactile information could be used as a second source of sensory information to improve the localization of a proprioceptive target.

The goal of this study was to investigate the influence of tactile information on the proprioceptive localization of the index finger in a motor context. In order to do so, we had participants perform a position-matching task in which they were asked to make reaches with the right index finger to a proprioceptive target (i.e., the opposite left index finger). No visual feedback of the hand was provided during reach execution and 30 or 300 Hz vibrotactile stimulations were applied on the left target index fingertip prior to movement onset. Trials in which no tactile vibration was delivered to the left index finger were also included. Constant *x*-errors and dispersion errors were measured and compared across all three experimental conditions. Constant *x*-errors represent the reach accuracy, that is to say how close the right reaching finger is from the left target finger; the smaller the constant *x*-error, the greater the reach accuracy. Dispersion errors refer to reach precision which reflect how consistent reach endpoints are when repeated; the smaller the dispersion error, the greater the reach precision.

We found that reach accuracy and precision, measured as constant and dispersion errors respectively, were both affected by the application of vibrotactile stimulations on the left target index fingertip. Indeed, both the constant and the dispersion errors were reduced when 30 or 300 Hz vibrations were delivered, as compared to the no vibration condition. Thus, it seems that cutaneous vibrations at either low or high frequencies provided the nervous system with additional (though slightly different) tactile information about the left index finger position. As a result, the spatial localization of the proprioceptive target was enhanced and both the accuracy and the precision of reaching were improved relative to the condition with no tactile stimulation. These results suggest that tactile information from the cutaneous vibrations is integrated with proprioceptive information about the position of the target index finger. In accordance with multisensory integration principles, the congruent proprioceptive and tactile information enhanced the finger proprioceptive localization, and ultimately improved proprioceptive reach performance.

The finding that both 30 and 300 Hz vibrotactile stimulations similarly improve reaching performance does not allow us to conclude about the specific contributions of Meissner and Pacinian corpuscles to touch-proprioceptive integration. According to previous studies, low- and high-frequency cutaneous vibrations appear to have distinct effects on proprioceptive

acuity [20,21]. Performance in a passive finger movement detection task was impaired when stimulations at 300 Hz were delivered to the finger. In contrast, the application of 30 Hz vibrations did not alter task performance. However, in our study we found similar results when either 30 or 300 Hz vibrotactile stimulation was applied to the proprioceptive target of the reach (i.e., the left index finger). Both high- and low-frequency tactile stimulations led to an improvement in reach accuracy and precision when pointing to the left index finger. These discrepancies might be explained by the fact that the tasks used in these studies were fundamentally different. Participants in Weerakkody's studies [20,21] performed a perceptual task in which they reported whether the movement imposed to their finger was a flexion or an extension. In contrast, in our study, participants were asked to localize a proprioceptive target and match its position by reaching with the opposite index finger. It has been proposed that somatosensory, and thus proprioceptive and tactile information is processed differently for perception and for action [27]. Similar to the two cortical processing streams described in the visual system [28], the "ventral" pathway is concerned with conscious somatosensory perception and object recognition while the "dorsal" pathway is relevant for guidance of action. The functional dissociation between the two somatosensory pathways has been established by studies in brain-damaged patients showing that they could perform motor actions towards somatosensory targets which were not consciously perceived [29–31]. These two separate somatosensory streams might explain why vibrotactile information is processed differently in perceptual and motor tasks. Nevertheless, it has been reported that separating the different tactile afferent fibers is challenging. It does not only depend on the stimulus frequency, but also on other parameters such as skin temperature [32]. Moreover, Meissner and Pacinian corpuscles are likely to have partially overlapping sensitivities, and thus detection thresholds which are relatively close to each other [19,32,33]. In the present study, it is therefore possible that the two vibrotactile frequencies delivered to the left target index finger might have activated both Meissner and Pacinian corpuscles. That could also explain why we did not observe difference between the 30 and 300 Hz vibration conditions.

In the present study, we found that tactile information provided on fingertip was integrated with proprioception, resulting in an improved spatial localization of the target fingertip during proprioceptive reaching. It could be that this improvement in spatial localization produced by the tactile stimuli was due to arousal enhancement related to the presence of an additional signal (i.e., the vibration). However, we found in a control experiment that putting the same vibration on the left shoulder did not improve reach precision (dispersion errors) as it did when the finger was vibrated, and while it produced changes to reach accuracy (constant *x*-errors), these were in the opposite manner as expected. Indeed, there was a decrease in accuracy rather than an increase as would be expected by increased arousal. Furthermore this effect was not consistent across the two vibration frequencies. Alternatively, enhanced spatial localization of the left target finger following vibrotactile stimuli could be explained by spatial attentional cueing effects. The vibration would act as a cue driving attention to the left index finger. If this was the case, a cue from another sensory modality (e.g. audition) delivered nearby the hand should improve fingertip localization as well. We believe that this is unlikely to account for our results since it has recently been shown that auditory cueing does not modulate hand localization accuracy [34]. Thus, we can rule out arousal and spatial attentional cueing effects.

Our effect results from a specific spatial integration of tactile and proprioceptive information. However, the exact mechanisms underlying this multisensory integration remain to be determined. According to the classic view of somatosensory processing, although both ascending through the dorsal column-medial lemniscal pathway, tactile and proprioceptive inputs remain segregated and are transmitted to distinct areas of the primary somatosensory cortex (S1) [35]. Somatosensory signals are not merged together until they reach higher-order

somatosensory areas, such as the posterior parietal cortex. This integration is thought to be mediated by area 5 in the intraparietal cortex, where both tactile and proprioceptive inputs converge [36]. However, electrophysiological recordings (mainly in areas 3b, 1 and 2) have provided evidence that some neurons in S1 respond to both tactile and proprioceptive signals [16,17,37]. These findings support an alternative but not exclusive hypothesis that multimodal interaction, and thus integration, between touch and proprioception might also occur at the level of S1, presumably in all sub-areas. Indeed, about half of S1 neurons, located in multi-modal areas 1 and 2 but also in the previously thought modality-specific areas 3a (proprioception) and 3b (cutaneous), showed responses to both proprioceptive and tactile stimuli [38]. Further research is needed to elucidate the mechanisms underpinning touch-proprioceptive integration and determine how tactile inputs influence the processing of proprioceptive information.

Acknowledgments

The authors would like to thank Romain Fournet for technical assistance.

Author Contributions

Conceptualization: Laura Mikula, Gunnar Blohm, Aarlenne Zein Khan.

Data curation: Sofia Sahnoun.

Formal analysis: Laura Mikula, Sofia Sahnoun, Laure Pisella, Aarlenne Zein Khan.

Investigation: Laura Mikula, Laure Pisella, Aarlenne Zein Khan.

Methodology: Laura Mikula, Gunnar Blohm, Aarlenne Zein Khan.

Writing – original draft: Laura Mikula, Aarlenne Zein Khan.

Writing – review & editing: Laura Mikula, Sofia Sahnoun, Laure Pisella, Gunnar Blohm, Aarlenne Zein Khan.

References

1. Knill DC, Pouget A. The Bayesian brain: the role of uncertainty in neural coding and computation. Trends Neurosci. 2004 Dec; 27(12):712–9. https://doi.org/10.1016/j.tins.2004.10.007 PMID: 15541511
2. O'Reilly JX, Jbabdi S, Behrens TEJ. How can a Bayesian approach inform neuroscience? Eur J Neurosci. 2012 Apr; 35(7):1169–79. https://doi.org/10.1111/j.1460-9568.2012.08010.x PMID: 22487045
3. Godfroy-Cooper M, Sandor PMB, Miller JD, Welch RB. The interaction of vision and audition in two-dimensional space. Front Neurosci. 2015; 9:311. https://doi.org/10.3389/fnins.2015.00311 PMID: 26441492
4. Hairston WD, Wallace MT, Vaughan JW, Stein BE, Norris JL, Schirillo JA. Visual localization ability influences cross-modal bias. J Cogn Neurosci. 2003 Jan 1; 15(1):20–9. https://doi.org/10.1162/089892903321107792 PMID: 12590840
5. van Beers RJ, Sittig AC, van der Gon Denier JJ. How humans combine simultaneous proprioceptive and visual position information. Exp Brain Res. 1996 Sep; 111(2):253–61. PMID: 8891655
6. van Beers RJ, Sittig AC, van der Gon Denier JJ. Localization of a seen finger is based exclusively on proprioception and on vision of the finger. Exp Brain Res. 1999 Mar; 125(1):43–9. PMID: 10100975
7. Warren JP, Santello M, Helms Tillery SI. Effects of fusion between tactile and proprioceptive inputs on tactile perception. PloS One. 2011 Mar 25; 6(3):e18073. https://doi.org/10.1371/journal.pone.0018073 PMID: 21464943
8. de Vignemont F, Ehrsson HH, Haggard P. Bodily illusions modulate tactile perception. Curr Biol CB. 2005 Jul 26; 15(14):1286–90. https://doi.org/10.1016/j.cub.2005.06.067 PMID: 16051171
9. Moberg E. The role of cutaneous afferents in position sense, kinaesthesia, and motor function of the hand. Brain J Neurol. 1983 Mar; 106 (Pt 1):1–19.

10. Helms Tillery SI, Flanders M, Soechting JF. Errors in kinesthetic transformations for hand apposition. Neuroreport. 1994 Dec 30; 6(1):177–81. PMID: 7703409
11. Rao AK, Gordon AM. Contribution of tactile information to accuracy in pointing movements. Exp Brain Res. 2001 Jun; 138(4):438–45. PMID: 11465741
12. Rincon-Gonzalez L, Naufel SN, Santos VJ, Helms Tillery S. Interactions between tactile and proprioceptive representations in haptics. J Mot Behav. 2012; 44(6):391–401. https://doi.org/10.1080/00222895.2012.746281 PMID: 23237463
13. Lackner JR, Dizio P. Rapid adaptation to Coriolis force perturbations of arm trajectory. J Neurophysiol. 1994 Jul; 72(1):299–313. https://doi.org/10.1152/jn.1994.72.1.299 PMID: 7965013
14. Gordon AM, Soechting JF. Use of tactile afferent information in sequential finger movements. Exp Brain Res. 1995; 107(2):281–92. PMID: 8773246
15. Rincon-Gonzalez L, Warren JP, Meller DM, Tillery SH. Haptic interaction of touch and proprioception: implications for neuroprosthetics. IEEE Trans Neural Syst Rehabil Eng Publ IEEE Eng Med Biol Soc. 2011 Oct; 19(5):490–500.
16. Cohen DA, Prud'homme MJ, Kalaska JF. Tactile activity in primate primary somatosensory cortex during active arm movements: correlation with receptive field properties. J Neurophysiol. 1994 Jan; 71 (1):161–72. https://doi.org/10.1152/jn.1994.71.1.161 PMID: 8158225
17. Prud'homme MJ, Cohen DA, Kalaska JF. Tactile activity in primate primary somatosensory cortex during active arm movements: cytoarchitectonic distribution. J Neurophysiol. 1994 Jan; 71(1):173–81. https://doi.org/10.1152/jn.1994.71.1.173 PMID: 8158227
18. Macefield VG. Physiological characteristics of low-threshold mechanoreceptors in joints, muscle and skin in human subjects. Clin Exp Pharmacol Physiol. 2005 Feb; 32(1–2):135–44. https://doi.org/10.1111/j.1440-1681.2005.04143.x PMID: 15730450
19. Talbot WH, Darian-Smith I, Kornhuber HH, Mountcastle VB. The sense of fluttervibration: comparison of the human capacity with response patterns of mechanoreceptive afferents from the monkey hand. J Neurophysiol. 1968 Mar; 31(2):301–34. https://doi.org/10.1152/jn.1968.31.2.301 PMID: 4972033
20. Weerakkody NS, Mahns DA, Taylor JL, Gandevia SC. Impairment of human proprioception by high-frequency cutaneous vibration. J Physiol. 2007 Jun 15; 581(Pt 3):971–80. https://doi.org/10.1113/jphysiol.2006.126854 PMID: 17412774
21. Weerakkody NS, Taylor JL, Gandevia SC. The effect of high-frequency cutaneous vibration on different inputs subserving detection of joint movement. Exp Brain Res. 2009 Aug; 197(4):347–55. https://doi.org/10.1007/s00221-009-1921-3 PMID: 19593554
22. Rossetti Y, Stelmach G, Desmurget M, Prablanc C, Jeannerod M. The effect of viewing the static hand prior to movement onset on pointing kinematics and variability. Exp Brain Res. 1994; 101(2):323–30. PMID: 7843319
23. Ernst MO, Bülthoff HH. Merging the senses into a robust percept. Trends Cogn Sci. 2004 Apr; 8 (4):162–9. https://doi.org/10.1016/j.tics.2004.02.002 PMID: 15050512
24. van Beers RJ, Sittig AC, van der Gon Denier JJ. Integration of proprioceptive and visual position-information: An experimentally supported model. J Neurophysiol. 1999 Mar; 81(3):1355–64. https://doi.org/10.1152/jn.1999.81.3.1355 PMID: 10085361
25. Sober SJ, Sabes PN. Flexible strategies for sensory integration during motor planning. Nat Neurosci. 2005 Apr; 8(4):490–7. https://doi.org/10.1038/nn1427 PMID: 15793578
26. van Beers RJ, Sittig AC, van der Gon Denier JJ. The precision of proprioceptive position sense. Exp Brain Res. 1998 Oct; 122(4):367–77. PMID: 9827856
27. Dijkerman HC, de Haan EHF. Somatosensory processes subserving perception and action. Behav Brain Sci. 2007 Apr; 30(2):189–201; discussion 201–239. https://doi.org/10.1017/S0140525X07001392 PMID: 17705910
28. Goodale MA, Milner AD. Separate visual pathways for perception and action. Trends Neurosci. 1992 Jan; 15(1):20–5. PMID: 1374953
29. Aglioti S, Beltramello A, Bonazzi A, Corbetta M. Thumb-pointing is humans after damage to somatic sensory cortex. Exp Brain Res. 1996 Apr; 109(1):92–100. PMID: 8740212
30. Paillard J, Michel F, Stelmach G. Localization without content. A tactile analogue of "blind sight". Arch Neurol. 1983 Sep; 40(9):548–51. PMID: 6615285
31. Rossetti Y, Rode G, Boisson D. Implicit processing of somaesthetic information: a dissociation between where and how? Neuroreport. 1995 Feb 15; 6(3):506–10. PMID: 7766853
32. Bolanowski SJ, Gescheider GA, Verrillo RT, Checkosky CM. Four channels mediate the mechanical aspects of touch. J Acoust Soc Am. 1988 Nov; 84(5):1680–94. PMID: 3209773

33. Mountcastle VB, LaMotte RH, Carli G. Detection thresholds for stimuli in humans and monkeys: comparison with threshold events in mechanoreceptive afferent nerve fibers innervating the monkey hand. J Neurophysiol. 1972 Jan; 35(1):122–36. https://doi.org/10.1152/jn.1972.35.1.122 PMID: 4621505

34. Bellan V, Gilpin HR, Stanton TR, Dagsdóttir LK, Gallace A, Lorimer Moseley G. Relative contributions of spatial weighting, explicit knowledge and proprioception to hand localisation during positional ambiguity. Exp Brain Res. 2017; 235(2):447–55. https://doi.org/10.1007/s00221-016-4782-6 PMID: 27778047

35. Mountcastle VB. The Sensory Hand: Neural Mechanisms of Somatic Sensation. Harvard University Press; 2005. 648 p.

36. Rizzolatti G, Luppino G, Matelli M. The organization of the cortical motor system: new concepts. Electroencephalogr Clin Neurophysiol. 1998 Apr; 106(4):283–96. PMID: 9741757

37. Weber DJ, London BM, Hokanson JA, Ayers CA, Gaunt RA, Torres RR, et al. Limbstate information encoded by peripheral and central somatosensory neurons: implications for an afferent interface. IEEE Trans Neural Syst Rehabil Eng Publ IEEE Eng Med Biol Soc. 2011 Oct; 19(5):501–13.

38. Kim SS, Gomez-Ramirez M, Thakur PH, Hsiao SS. Multimodal interactions between proprioceptive and cutaneous signals in primary somatosensory cortex. Neuron. 2015 Apr 22; 86(2):555–66. https://doi.org/10.1016/j.neuron.2015.03.020 PMID: 25864632
